# Development of Resazurin-Based Assay for Rapid Evaluation of Sodium Hypochlorite Tolerance in *Salmonella*

**DOI:** 10.3390/foods15061086

**Published:** 2026-03-20

**Authors:** Feng Liu, Jiele Ma, Yingping Xiao, Wen Wang, Yangtai Liu, Qingli Dong, Xingning Xiao

**Affiliations:** 1 College of Health Science and Engineering, University of Shanghai for Science and Technology, Shanghai 200093, China; lf18389194946@163.com (F.L.); usstlyt@163.com (Y.L.); 2 MOA Laboratory of Quality & Safety Risk Assessment for Agro-Products (Hangzhou), Institute of Agro-Product Safety and Nutrition, Zhejiang Academy of Agricultural Sciences, Hangzhou 310018, China; jlma163@163.com (J.M.); ypxiaozj@hotmail.com (Y.X.); 3 Zhejiang Provincial Key Laboratory of Biometrology and Inspection & Quarantine, China Jiliang University, Hangzhou 310018, China; ww_hi1018@163.com

**Keywords:** chlorine tolerance, MIC, *Salmonella*, sanitizer, hypochlorite

## Abstract

Sodium hypochlorite (NaClO) is frequently utilized in food processing. More than 90% of *Salmonella* spp. isolates from poultry supply chains exhibited tolerance to NaClO, with MIC values exceeding 256 mg/L. Exposure to NaClO disinfection may lead to the emergence of bacterial tolerance to chlorine, which is frequently associated with antibiotic cross-resistance. This work employed a resazurin-based assay for rapid evaluation of the NaClO chlorine tolerance of *Salmonella*. The results were compared to the broth microdilution method for assessing bacterial tolerance. At the initial inoculum of 10^7^ CFU/mL, NaClO tolerance was successfully identified via colorimetry within 2 h. Notably, the fluorescence-based evaluation yielded significant results even sooner, showing a marked increase in intensity within 1 h of resazurin incubation. Even with an inoculum of 10^5^ CFU/mL, the resazurin-based method determines NaClO tolerance in just 6 h. Conversely, traditional broth microdilution requires an overnight culture to manifest sufficient turbidity for optical density monitoring. Furthermore, the broth microdilution method revealed NaClO tolerance (MIC > 256 mg/L) in 1.6% (1/64) of the *Salmonella* isolates. The modified resazurin assay, by contrast, detected tolerance in 6.3% (4/64) of isolates. The reference that differentiates between resistant and sensitive strains was 3.2 × 10^5^ RFU. When the strains exhibited an MIC value of 256 mg/L, the fluorescence intensity varied from around 1.2 × 10^5^ to 4 × 10^5^ RFU, reflecting inactivation effects at practical chlorine concentrations. This methodology is recognized as a rapid, high-throughput, and quantitative screening approach for assessing bacterial chlorine resistance.

## 1. Introduction

*Salmonella* is responsible for approximately 70–80% of foodborne disease outbreaks in China [1,2,3]. Chlorine sanitizers, including sodium hypochlorite (NaClO), sodium dichloroisocyanurate, and chloramine, are widely utilized in the food industry to reduce microbial load [4,5]. The World Health Organization (WHO) recommends the use of a 1000–5000 mg/L NaClO solution (pH 8–10) for disinfecting domestic surfaces [6,7,8]. During the spin-chill process in poultry processing, chicken carcasses are immersed in cold water with 50 mg/L free chlorine added to maintain a near-neutral pH (6.5~7) [9]. This serves to both maximize free chlorine concentration and minimize disinfection byproduct formation [9]. With increasing NaClO concentration (0–200 mg/L), the viable count of pathogenic bacteria decreased significantly within a 5 min treatment [10]. Chlorine is also used to prevent cross-contamination in wash tanks. The effectiveness of chlorine is dependent not only on the concentration of chlorine but also on the chemical composition of the chill water. Chlorine effectiveness was diminished by the inorganic and organic components present in chilled water because chlorine reacted not only with microorganisms but also with the inorganic and organic materials [11,12]. Nonetheless, throughout the sanitization process, bacteria can survive despite the presence of residual disinfectants, thereby earning the designation of being tolerant [13,14]. Extended exposure to disinfectants or the excessive application (e.g., excessively high concentration, excessive dosage) of chlorine disinfectants presents a worldwide threat of antibiotic-resistant bacterial development, endangering human health. This is attributed to the generation of selective pressure, which increases the likelihood of horizontal gene transfer (HGT) within and between bacterial genera.

Tolerance refers to the capacity of a bacterial population to survive transient exposure to lethal concentrations of an antimicrobial, which is characterized by a significantly reduced killing rate, even in the absence of changes in MIC [5,15]. A total of 1722 publications were available on the Web of Science under the topic ‘chlorine-resistant bacteria’, with a significant increase in the past five years [5]. A previous study reported that *Salmonella* isolates (*n* = 53) showed a broader MIC range of 32–512 mg/L against chlorine disinfectants [16]. Despite apprehensions regarding the escalating resistance of foodborne pathogens to chlorine disinfectants, no universally recognized approach is available for evaluating chlorine-tolerant bacteria [5,17]. Prior research has employed broth microdilution, inhibitory zone, logarithmic elimination rate, and membrane damage methods to evaluate bacterial tolerance to chlorine [14,17]. The broth microdilution is the standard method established by the Clinical Laboratory Standards Institute (CLSI), which is a widely utilized technique for evaluating bacterial resistance to antibiotics or disinfectants [18]. The broth microdilution approach with turbidity measurement is appropriate for comparing chlorine tolerance among isolates; however, it cannot reflect the inactivation effect at the same minimal inhibitory concentration (MIC) values [5]. Kampf proposed isolates that are inadequately killed by below use concentrations of a disinfectant solution in suspension tests could be considered tolerant and potentially resistant; moreover, regardless of changes in the MIC value, any isolate or strain should be considered resistant if it demonstrates a significantly elevated minimum bactericidal concentration (MBC), provided the concentration used in the MBC test complies with standard practice and achieves the commonly accepted minimum log_10_ reduction [5,15]. Current traditional protocols, most notably the broth microdilution method, are primarily designed to evaluate the MIC value by detecting visible turbidity. However, this process necessitates an 18–24 h incubation period, which is often too slow for rapid decision-making in food processing or clinical environments. Furthermore, even when MIC values remain identical, significant phenotypic variability may persist among bacterial isolates, complicating the detection of subtle shifts in tolerance levels [5].

Resazurin is a water-soluble, nontoxic, and non-fluorescent blue dye. When reduced by metabolically active bacteria, it is converted into resorufin, a pink and strongly fluorescent molecule that accumulates in the culture medium [19,20]. Resazurin-based assays, including AlamarBlue^®^ (AB) and PrestoBlue™ (PB), operate on the principle of reducing resazurin to its highly fluorescent derivative, resorufin. The reduction of resorufin correlates with the quantity of metabolically active cells and can be assessed by spectrometry or fluorometry [19,20]. The resazurin reduction assay primarily detects metabolically active cells and may not respond to dormant or viable but non-culturable (VBNC) cells [21]. The various metabolic capabilities may influence the disinfection resistance of bacteria. PrestoBlue can be decreased by NADPH, NADH, and cytochromes [22]. The initial phase of this reduction results from the elimination of a loosely attached oxygen atom from the nitrogen of the phenoxazine nucleus. The transformation to pink resorufin is irreversible in the presence of ambient oxygen and is predominantly unaffected by reduction potential and oxygen concentration. The second stage of reduction to a colorless state is reversible by ambient oxygen [23]. Resazurin exhibits considerable stability in a cell-free culture medium, although it undergoes fast reduction in the presence of viable cells. Various reductases, including diaphorases and NADPH dehydrogenase, may utilize resazurin as an electron acceptor, facilitating the reduction of resazurin to resorufin [23]. In our previous study, the NAD^+^/NADH ratio increased by 14.36 fold in the susceptible strain and by 4.19 fold in the tolerant strain. This marked elevation suggests a significantly more oxidizing intracellular redox state in the sensitive strain. Resazurin has been utilized to evaluate planktonic bacterial survival, stress tolerance, and bacterial contamination [19]. It has been widely utilized, particularly for antibacterial evaluations and studies on bacterial resistance [19,24]. It was also used to evaluate NaClO disinfection efficacy on biofilms of several clinically relevant pathogenic bacteria [25]. At present, few studies investigated the resazurin-based assay for rapid evaluation of sodium hypochlorite tolerance in *Salmonella*.

This study aimed to develop a resazurin-based assay for evaluating NaClO tolerance in *Salmonella*, using changes in colorimetric and fluorescence intensity as indicators of bacterial response. The method was applied to assess NaClO tolerance across 64 *Salmonella* isolates. The resazurin-based assay, a rapid and high-throughput screening method, could facilitate the systematic surveillance of disinfectant resistance trends across temporal and geographic scales.

## 2. Materials and Methods

### 2.1. Bacterial Inoculum

*Salmonella enteritidis* CVCC 1806 (*S. enteritidis* CVCC 1806) is preserved and distributed by the China Veterinary Culture Collection (CVCC), a national repository for veterinary-related microorganisms in China. It is originally isolated from poultry and employed as a quality control strain in validation studies for detection methods, disinfectant protocols, and antimicrobial susceptibility testing [26]. Resazurin-based assay was developed for evaluation of NaCIO tolerance through using *S. enteritidis* CVCC 1806. Then the results of NaCIO tolerance in 64 *Salmonella* strains isolated from the poultry supply chain were compared with the broth microdilution method. All strains were stored in brain heart infusion (BHI, Becton Dickinson, East Rutherford, NJ, USA) broth containing 20% glycerol at −80 °C until use. The strains were separately incubated in BHI at 37 °C for 24 h. Bacterial cells were collected by centrifugation at 8000 rpm for 5 min, washed 3× with 0.85% (*w*/*v*) sterile saline, and suspended in 0.85% (*w*/*v*) sterile saline at 10^9^ CFU/mL. Appropriate 10-fold dilutions were prepared in sterile Mueller–Hinton broth (MH; Becton Dickinson) and plated onto xylose lysine Tergitol 4 (XLT4; Becton Dickinson) agar to enumerate viable cells in the inoculum.

### 2.2. Reagents Preparation

The commercial NaClO stock solution (Sangon Biotech, Shanghai, China) was used in this study. The available chlorine content of the original NaClO solution was 5.68% (56,800 mg/L, pH 12), which was subsequently diluted to target working concentrations for all assays [14]. Chlorine concentrations were determined using a Palintest ChlorSense meter (Gateshead, UK). PrestoBlue, a commercial resazurin solution (Invitrogen, Carlsbad, CA, USA, CAT#P50201), was used as the enzymatic substrate for cell viability assays following the manufacturer’s instructions.

### 2.3. Microplate Preparation

Chlorine Tolerance Assays. Chlorine tolerance was evaluated in 96-well microtiter plates. Sodium hypochlorite (NaClO) working solutions, with free chlorine concentrations ranging from 16 to 1024 mg/L, were prepared via two-fold serial dilution of the stock solution in sterile tubes. Subsequently, 100 µL of the bacterial suspension was inoculated into each well containing an equal volume of the disinfectant, resulting in theoretical final concentrations of 8 to 512 mg/L in the reaction mixture. To ensure accuracy, the free chlorine concentrations were verified using a calibrated Palintest ChlorSense meter (DPD standard method). Following a 30-min exposure at room temperature, the oxidative reaction was quenched by adding 20 µL of 2.5% Na_2_S_2_O_3_ and incubating for 10 min. The pH and residual free chlorine levels of the reaction mixtures were quantified and are summarized in Table S1. To maintain consistency with numerical representations in previous literature and ensure the continuity of the dilution series, the free chlorine concentrations of the NaClO working solutions were utilized to report the MIC values. The methodology was modified from the protocol established by previous studies to better achieve the aims of the current investigation [27,28].

### 2.4. NaClO Tolerance Determination Using Broth Microdilution Method

Moreover, 96-multiwell plates were prepared as described in Section 2.3. Microplates were incubated at 37 °C. The turbidity of the wells was visually recorded. Cell growth in the plates was also quantified by measuring the optical density at 544 nm in the turbidity test. The MIC of NaClO was defined as the lowest concentration at which no color changes occurred (OD_544_ < 0.2). Bacterial cultures without NaClO served as the positive control, while sterile MH broth was used as the negative control. All experiments were performed in triplicate on separate days.

### 2.5. NaClO Tolerance Determine Using Resazurin Assay

**Figure 1:**
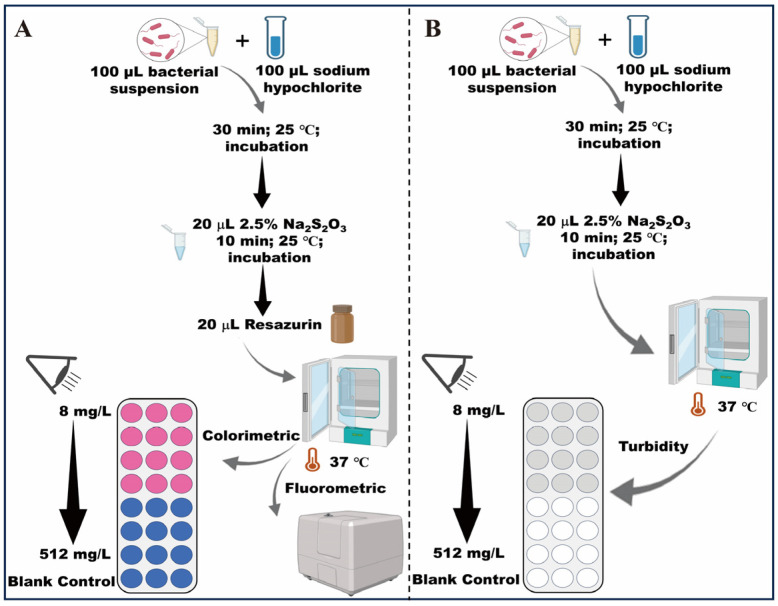
Development evaluation method of sodium hypochlorite tolerance in *Salmonella*: (**A**) flowchart of resazurin-based assay based on colorimetric and fluorescence intensity changes; (**B**) flowchart of broth microdilution method based on turbidity changes.

The assay was performed in MH broth. 96-well plates were made as outlined in Section 2.3. After neutralization of chlorine disinfectants by Na_2_S_2_O_3_, the pH of the solution becomes close to neutral. Then, a total of 20 μL of PrestoBlue resazurin solution was added to each well. The samples were subsequently incubated at 37 °C. Bacterial suspensions without NaClO served as the positive control, and sterile MH broth was used as the negative control. Color changes in the wells were initially monitored via visual inspection and subsequently quantified using a FLUOstar microplate reader. The blue was regarded as a lack of metabolic activity. The pink was taken as an indication of metabolic activity. The presence of a purple in the well was seen as a residual outcome, indicating some metabolic activity; however, an extended incubation period facilitated the transition of the purple color to pink [19]. Upon seeing a colorimetric change, the reduction of resazurin to resorufin was quantified using the FLUOstar microplate reader (Agilent, Santa Clara, CA, USA; BioTek Cytation 5, Shoreline, WA, USA) at excitation and emission wavelengths of 544 nm and 590 nm, respectively. Having validated the suitability of resazurin for assessing NaClO tolerance in *Salmonella*, we proceeded to optimize all assay parameters for subsequent screening experiments. These parameters included the inoculum size (10^1^~10^7^ CFU/mL) and incubation temperature (25 and 37 °C). All experiments were performed in triplicate on separate days. The evaluation method of NaClO tolerance is shown in Figure 1.

### 2.6. Statistical Analysis

GraphPad Prism 9.0 software was used for data analysis. The *t*-test was used for inter-group comparisons (*p* > 0.05 was considered non-significant). Data were presented as mean ± standard deviation.

## 3. Results and Discussion

### 3.1. Evaluation of NaClO Tolerance in Colorimetric Changes

**Figure 2:**
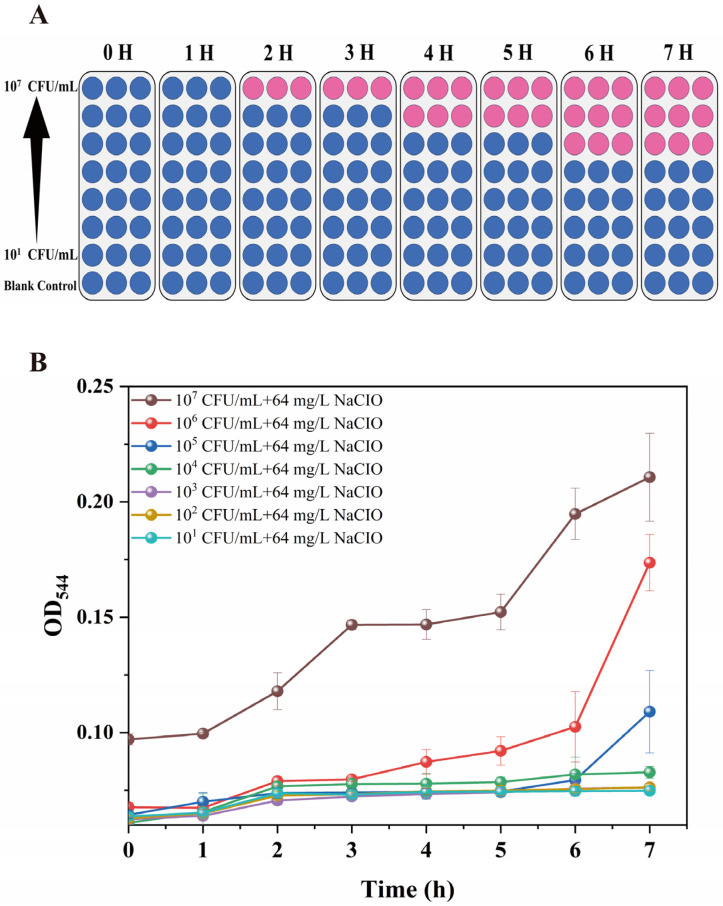
Visual observation time of sodium hypochlorite tolerance in *S. enteritidis* CVCC 1806 at a range of bacterial concentrations between 10^1^ and 10^7^ CFU/mL. The final concentrations of the NaClO solution were 64 mg/L. (**A**) NaClO tolerance in colorimetric; (**B**) NaClO tolerance by optical density measurements.

In order to prove the correlation between the resazurin reduction and the number of viable cells, we tested a range of bacterial concentrations between 10^1^ and 10^7^ CFU/mL in the 96-well microplate system (Figure 1 and Figure 2). Our resazurin-based method was capable of detecting *Salmonella* concentrations ranging from 10^5^ to 10^7^ CFU/mL within 6 h, with NaClO tolerance being determined by observable colorimetric changes (Figure 2A). The colorimetric change is readily observable in 2 h at a high inoculum level of 10^7^ CFU/mL. A clear colorimetric change was observed within 4 h at a concentration of 10^6^ CFU/mL. The colorimetric change becomes readily apparent after 6 h when using a low inoculum of 10^5^ CFU/mL (Figure 2A). The broth microdilution method, which relies on naked eye observation or optical density measurements, requires an overnight culture to monitor turbidity changes (Figure 2B). Generally, it requires overnight culturing for 18 to 24 h to monitor the turbidity changes. The resazurin assay has advanced significantly in terms of detection speed. When the MIC is identical, there is high variability in the results between parallel samples [21]. The broth microdilution method is applicable within specific concentration ranges; it is not suitable when cell lysis occurs. The dye acts as an intermediate electron acceptor in the electron transport chain without interference with the normal transfer of electrons. As the indicator dye accepts electrons, it changes from the oxidized, non-fluorescent, blue state to the reduced, fluorescent, pink state. Resazurin reduction may signify an impairment of cellular metabolism [19,29,30]. Additionally, we conducted multiple trials to determine the best incubation temperature for this assay in both microplates. We chose 37 °C, as this temperature markedly decreased the incubation duration for the experiment, particularly when contrasted with findings obtained at 25 °C (Figure S1).

### 3.2. Evaluation of NaClO Tolerance in Fluorescence Intensity Changes

**Figure 3:**
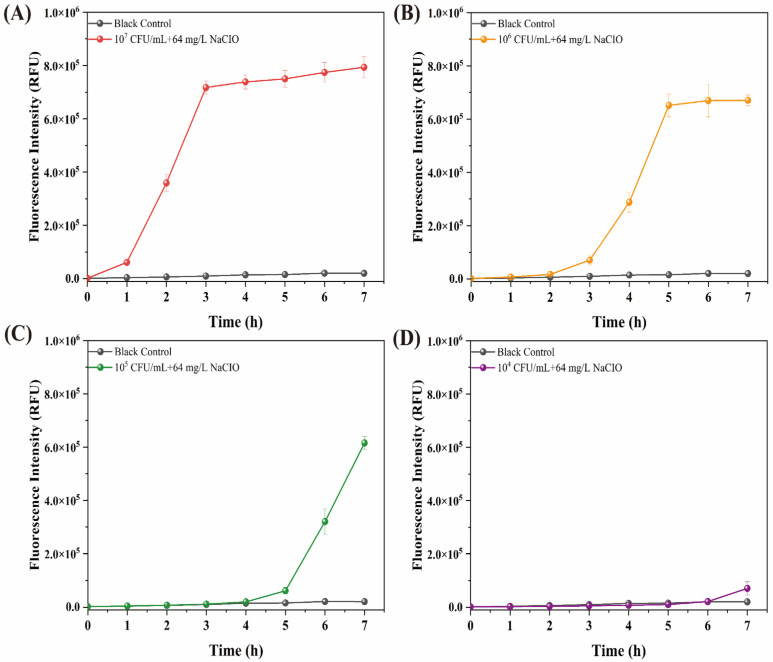
Fluorescence intensity changes in sodium hypochlorite tolerance in *S. enteritidis* CVCC 1806 at a range of bacterial concentrations between 10^4^ and 10^7^ CFU/mL. Bacterial concentrations at (**A**) 10^7^ CFU/mL; (**B**) 10^6^ CFU/mL; (C) 10^5^ CFU/mL; (**D**) 10^4^ CFU/mL.

The NaClO tolerance was evaluated using fluorescence intensity, which significantly increased (approximately 6 × 10^5^ RFU) with the administration of the resazurin reagent for 1 h at a high initial inoculum level of 10^7^ CFU/mL (Figure 3). When utilizing the colorimetric changes as the indicator of NaClO tolerance, the changes need to be observed after 2 h. The results showed that the fluorescence detection was more rapid, offering a 1 h advantage compared to the colorimetric assay. Additionally, the fluorescence intensity change is readily observable in 3 h at an inoculum level of 10^6^ CFU/mL. A clear fluorescence intensity change was observed within 5 h at a concentration of 10^5^ CFU/mL. The fluorescence intensity is 7 × 10^5^ RFU in 7 h at a low inoculum level of 10^4^ CFU/mL. For the broth microdilution method, no turbidity change was observed in *S. enteritidis* CVCC 1806 (Figure 2B). We chose fluorescence intensity variations rather than absorbance alterations as the readout parameter due to reduced background signals during fluorescence intensity changes. Additionally, in an environment where only an ultraviolet/visible spectrophotometer is accessible, the utilization of the resazurin test remains feasible [25]. The nontoxicity of resazurin is beneficial as it facilitates dynamic measurements [21]. It is noted that resazurin is low in toxicity, rather than nontoxic to cells. The cytotoxicity of resazurin is contingent upon its concentration and the duration of incubation with cells [31,32].

### 3.3. NaClO Tolerance Evaluation in Salmonella Isolates

In the broth microdilution method, tolerance to NaClO at a minimum inhibitory concentration (MIC) above 128 mg/L was observed in 98.4% (63 out of 64) of our *Salmonella* isolates. Sensitive to NaClO at MIC = 128 mg/L was observed in 1.6% (1 out of 64). In the resazurin-based experiment, tolerance to NaClO at a MIC above 128 mg/L was observed in 100%. The results indicate that the resazurin method detects a greater number of resistant strains compared to the MIC method, with a false resistance rate of 1.6% (Table 1). The resazurin assay has been widely used as a rapid screening test for assessing bacterial antibiotic resistance. For example, in the study of Jia et al. [24], a resazurin-based assay was evaluated for the rapid detection of polymyxin-resistant Gram-negative bacteria. The sensitivity and specificity of the test compared to the broth microdilution method were 100 and 99%, respectively. Rakhmawatie et al. [33] evaluated the performance of the resazurin microplate assay for antimycobacterial screening and found that the MIC values against *M. smegmatis* were interpreted 1 h after adding resazurin. While the resazurin assay is an excellent screening tool, its threshold parameters need careful calibration to align with gold-standard MIC results. It should be noted that the bacterial strains evaluated in this study exhibited a high prevalence of NaClO resistance. Consequently, the performance comparison between the resazurin assay and the standard broth microdilution method is predominantly representative of a resistant population. These findings may not fully capture the assay’s sensitivity in detecting hypochlorite susceptibility within a more diverse strain collection that includes a broader proportion of sensitive isolates. For instance, large-scale validation of resazurin-based assays, such as the work by Jia et al. [24] on polymyxin-resistant Gram-negative bacteria involving over 253 clinical isolates and 50 sensitive controls per species, highlights the necessity of extensive libraries for diagnostic robustness [24,33]. Future research incorporating a wider range of *Salmonella* serotypes and environmental isolates with varying susceptibility profiles is warranted to further validate the generalizability of this rapid screening framework.

**Figure 4:**
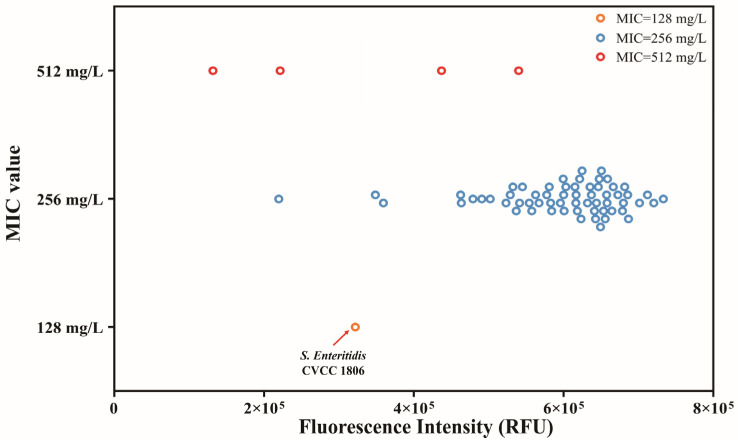
Fluorescence intensity changes in sodium hypochlorite tolerance in *S. enteritidis* CVCC 1806.

The reference that differentiates between resistant and sensitive strains was 3.2 × 10^5^ RFU. The mean fluorescence intensity values for tolerance (MIC = 256 mg/L) and tolerance (MIC = 512 mg/L) were 5.9 × 10^5^ RFU and 3.3 × 10^5^ RFU, respectively, and this difference was statistically significant (*p* < 0.05). Among the strains with an identical MIC value of 256 mg/L, fluorescence intensity varied from roughly 1.2 × 10^5^ to 4 × 10^5^ RFU, reflecting inactivation effects at practical chlorine concentrations (Figure 4). The majority of *S. typhimurium* (65.6%) isolated from carcass washing exhibited MIC at 80 mg/L. 68.95% of isolates exhibited MBC at 80 mg/L for chlorine. *S. typhimurium* isolated from the environmental samples exhibited similar MIC and MBC values for chlorine, while the highest proportion was exhibited at 40 mg/L, and it was 41.1% and 54.5%, respectively [34,35]. Several limitations of this study should be acknowledged. First, the sensitivity of the resazurin assay is inherently time-dependent. While our protocol was optimized for a rapid 6 h readout using an initial inoculum of 5–7 log CFU/mL, its lower limit of detection is constrained by incubation time. The relationship between lower bacterial loads and incubation time, or the development of a dynamic model, warrants further investigation. Second, at high bacterial densities, prolonged incubation may trigger the secondary reduction of resorufin into hydroresorufin, leading to signal saturation and a potential underestimation of metabolic activity. Third, as a metabolism-based indicator, the assay may fail to detect dormant or viable but non-culturable (VBNC) populations, which could lead to an underestimation of bacterial survival under certain stress conditions. Finally, the diversity of the *Salmonella* collection was relatively limited. Further validation using a broader panel of strains, including more sensitive isolates and diverse serotypes, is warranted to confirm the generalizability and robustness of this method for broader diagnostic applications.

The bactericidal activity of NaClO is pH-dependent, as the ratio of hypochlorous acid (HClO) to hypochlorite ion (ClO^−^) shifts with pH. At neutral pH, HClO is the dominant active species, whereas ClO^−^ becomes more prevalent and stable in alkaline conditions (typically pH > 10) [11]. A review of established protocols for chlorine MIC testing reveals that pH adjustment is generally not performed [27,28,36]. To ensure our results are comparable with existing literature on *Salmonella* and *Klebsiella pneumoniae* tolerance, we maintained consistency with these established methodologies [16,36]. For example, according to the report from Gao et al. [28], bacterial suspension was added to a 96-well microplate containing 50 μL of chlorine disinfectant per well, resulting in final concentrations of 1–512 mg/L. Additionally, Wu et al. [16] found that *Klebsiella pneumoniae* was more tolerant among Gram-negative strains to chlorine disinfectants, followed by *Salmonella* and *Escherichia coli*. The lack of standardized pH controls in chlorine susceptibility testing complicates the comparison of tolerance trends across studies. Future studies will prioritize the optimization of pH-controlled procedures.

## 4. Conclusions

An evaluation method of sodium hypochlorite tolerance in *Salmonella* using resazurin was developed and could be used as a screening test. The best conditions for the resazurin method were incubated at 37 °C, with an initial inoculum level at 7 log CFU/mL, and it reacted for 2 h. The principle of this test is based on the visual and fluorescence intensity changes. The results of NaClO tolerance can be finalized within one hour. Such a time-critical tool is essential for implementing precision disinfection strategies, minimizing chemical waste, and reducing the selective pressure for antimicrobial resistance. Our method could support the risk characterization and the development of precision disinfection strategies, minimizing both chemical waste and the selective pressure for resistance. In the future, continuous efforts are needed in technology optimization, standard unification, and multidisciplinary cooperation to address the increasingly serious issue of chlorine tolerance and provide reliable data support for control measures and policy decisions.

## Figures and Tables

**Table 1:** NaCIO Tolerance in *Salmonella* isolates from the poultry supply chain via the resazurin method and broth microdilution method (*n* = 64).

NaCIO Tolerance	Broth Microdilution	* Modified Resazurin Assay
Percentages of sensitive	1.6% (1/64)	0
Percentages of resistant	98.4% (63/64)	100%
Percentages of false sensitive	_	_
Percentages of false resistant	_	1.6% (1/64)

* Final bacterial concerntraion level at 10^5^ CFU/mL in the 96-multiwell plates.

## Data Availability

The original contributions presented in this study are included in the article/Supplementary materials. Further inquiries can be directed to the corresponding authors.

## Supplementary Materials

The following supporting information can be downloaded at https://www.mdpi.com/article/10.3390/foods15061086/s1. Figure S1: Fluorescence changes in sodium hypochlorite tolerance of *Salmonella* at incubation temperatures of 25 °C and 37 °C. Table S1: Characterization of free chlorine concentration and pH values in experimental systems.
