# Supplementary material for: Development of Resazurin-Based Assay for Rapid Evaluation of Sodium Hypochlorite Tolerance in Salmonella
Source: Foods. 2026 Mar 20;15(6):1086. doi: 10.3390/foods15061086 (PMC13026011; doi:10.3390/foods15061086)
Supplement: Supplementary file 1 [file foods-15-01086-s001.zip › foods-4183757-supplementary.pdf]

Figure S1. Fluorescence changes in sodium hypochlorite tolerance of *Salmonella* at incubation temperatures of 25 °C and 37 °C.

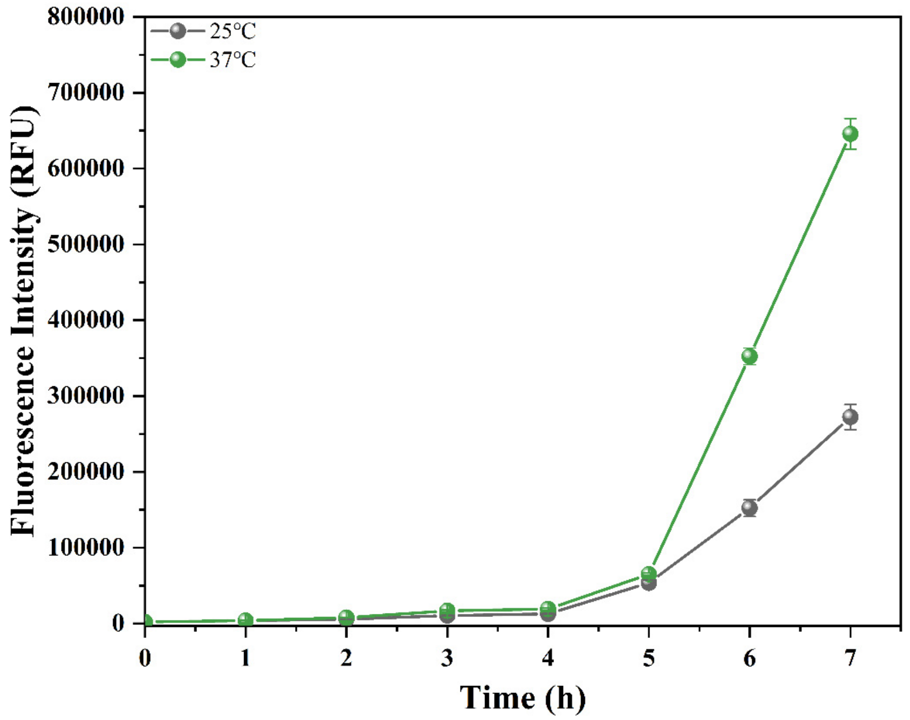

Table S1 Characterization of free chlorine concentration and pH values in experimental systems

Free Chlorine of the initial NaClO working Concentration (mg/L)	pH of NaClO working solutions	Free Chlorine in Reaction Mixture (mg/L) *	pH of Reaction Mixture
16	7.39	<0.1	7.31
32	7.44	0.18	7.33
64	7.55	0.37	7.35
128	7.62	2.3	7.37
256	7.88	41.2	7.41
512	8.14	76.9	7.43
1024	8.53	219	7.48

*Note: Measured immediately after mixing the NaClO working solution with the bacterial suspension in the culture media, representing the actual initial exposure concentration after accounting for instantaneous chlorine demand.
